# Adult-Onset Coats Disease With Rapid Progression to Full-Thickness Macular Hole: A Case Report

**DOI:** 10.7759/cureus.112529

**Published:** 2026-07-12

**Authors:** Bartlomiej Cwikla, Dawid Bartosik, Jakub Szkuta, Bartosz Starzynski, Alina Keska, Michal Kieres, Klaudia Wojcik, Anna Kowalczyk, Michal Sobczak, Michalina Loson-Kawalec, Piotr Madejczyk, Wiktoria Lazienka, Aleksander Tabor

**Affiliations:** 1 Faculty of Medicine, University of Opole, Opole, POL; 2 General Practice, Ministry of the Interior and Administration (MSWiA) Hospital, Opole, POL; 3 Faculty of Medicine, Wrocław Medical University, Wrocław, POL

**Keywords:** adult-onset, atypical coats disease, coats disease, full-thickness macular hole, pars plana vitrectomy, retinal telangiectasia

## Abstract

We present an atypical case of adult-onset Coats disease in a 39-year-old woman with rapid progression and the development of a full-thickness macular hole despite multimodal non-surgical treatment.

The patient presented to the emergency department with sudden deterioration of vision in the left eye lasting three days. Fundus examination revealed peripapillary hemorrhages, vitreous condensations, and focal exudation in the inferotemporal quadrant. Optical coherence tomography showed retinal thickening, intraretinal and subretinal fluid, and an epiretinal membrane. Fluorescein angiography demonstrated abnormal peripheral vessels with surrounding non-perfusion. The patient was treated with laser photocoagulation, intravitreal bevacizumab injections, and cryotherapy. Despite temporary stabilization, follow-up examinations showed progression of exudative and hemorrhagic changes, with subsequent formation of a full-thickness macular hole. Pars plana vitrectomy was performed. Postoperative optical coherence tomography confirmed closure of the macular hole.

This case highlights that adult-onset Coats disease may present with an atypically aggressive course and that conservative treatment may be insufficient in selected adult patients.

## Introduction

Coats' disease was first described in 1908 by George Coats, a Scottish ophthalmologist, as a non-hereditary retinal vascular pathology associated with exudation. In 1955, the knowledge regarding this disorder was expanded - Reese presented that the essence of the disease is retinal telangiectasia, which leads to progressive exudation and retinal detachment [1-3].

Epidemiologically, the occurrence of Coats' disease is estimated at 0.09 per 100,000 individuals [4]. Typically, it appears unilaterally in young men, and the rate of progression is slow; however, cases of this disorder occurring bilaterally, in adults, and in women have also been described [2,5].

The fundamental pathogenetic process of Coats' disease is idiopathic endothelial dysfunction leading to the breakdown of the blood-retinal barrier and progressive damage to vessel walls [4]. Pathology primarily involves the temporal and inferior retinal quadrants [3]. Progressive degeneration of the vascular wall and loss of pericytes result in the formation of aneurysms, followed by ischemia and subretinal or intraretinal exudation rich in lipids - as a result, the retina becomes thickened [2]. All these changes can lead to exudative retinal detachment [6].

In patients with Coats' disease, the anterior segment of the eye typically shows corneal edema, megalocornea, shallow anterior chamber, anterior chamber cholesterolosis, areas of capillary ischemia, iris heterochromia, and cataracts [1]. Patients often report pain, nystagmus, strabismus, or vision loss. Symptoms similar to those of Coats' disease may be observed in conditions such as inflammation, infection, degenerative retinal changes, or after radiotherapy [2]. There are cases where Coats' disease was asymptomatic and was detected only during fundus examination in elderly patients [1].

To diagnose Coats' disease, a detailed clinical evaluation and ophthalmoscopic examination should be performed [7]. The characteristic changes observed are retinal telangiectasias, which are present in all cases. These are called "bulb telangiectasias" [1]. Imaging tests are an essential part of differential diagnosis, diagnosis, and treatment [2]. The differential diagnosis of Coats disease includes other exudative retinopathies that present with a similar clinical course and retinal findings [1].

The primary goal of treatment in Coats disease is the elimination of telangiectatic vessels, which leads to the resolution of subretinal and intraretinal exudation. In patients with Coats disease, the aim is to preserve visual acuity and prevent the development of complications. Treatment should be initiated when disease progression is observed or when symptoms begin to impair daily functioning [2]. The choice of treatment depends on the stage of the disease according to the classification proposed by Shields et al., as well as its correlation with clinical symptoms [3].

The following case shows a rare form of Coats disease. It differs from the typical presentation in the patient’s age and sex, the fast progression with worsening retinal changes causing vision loss, and the location of the telangiectasia.

## Case presentation

A 39-year-old female patient presented to the emergency department (ED) because of sudden deterioration of vision in the left eye (OS), which had persisted for three days. Initial examination showed best-corrected visual acuity (BCVA) of 1.0 in the right eye (OD) and 0.1 in the OS. Intraocular pressure was within normal limits. Anterior segment examination of both eyes showed no abnormalities. The medical history was significant only for Hashimoto’s disease.

Fundus examination of the OS revealed peripapillary hemorrhages, an evolving macular hole, and vitreous condensations along the superotemporal vascular arcade. In addition, an area of exudation was found in the inferotemporal quadrant, suggesting a vascular lesion.

**Figure 1:**
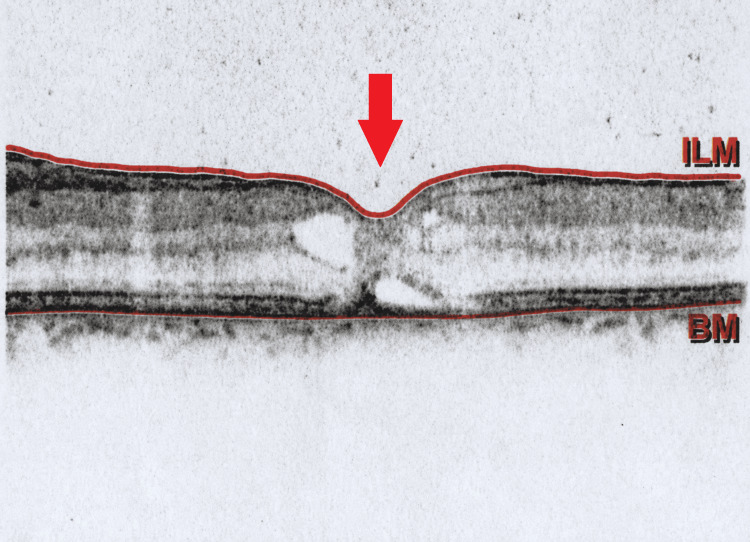
Optical coherence tomography of the left eye demonstrating retinal thickening with the presence of intraretinal and subretinal fluid (arrow).

**Figure 2:**
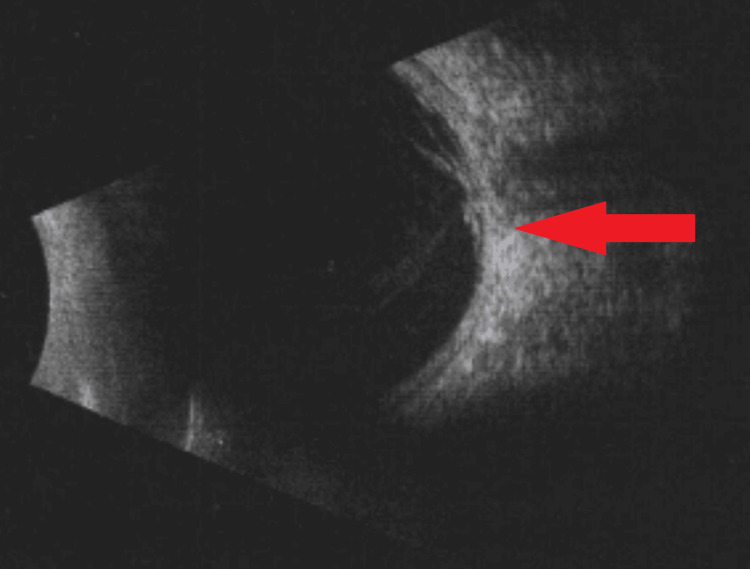
B-scan ultrasonography (USG) of the left eye showing complete retinal attachment in all quadrants (arrow).

Optical coherence tomography (OCT) showed retinal thickening, the presence of intraretinal and subretinal fluid, and an epiretinal membrane (ERM), indicating early structural abnormalities within the macula (Figure 1). B-scan ultrasonography confirmed that the retina was attached in all quadrants, with coexisting vitreoretinal traction (Figure 2).

Further diagnostics included fluorescein angiography, which revealed irregular hyperfluorescence in the peripheral inferotemporal area, surrounded by regions of non-perfusion, corresponding to the presence of pathological vessels. The patient was qualified for urgent laser therapy, including photocoagulation of the vascular proliferation focus in the inferotemporal quadrant. Due to the clinical presentation, treatment was supplemented with an intravitreal injection of the anti-vascular endothelial growth factor (anti-VEGF) agent, bevacizumab, performed a few days after laser photocoagulation.

At the follow-up visit after one month, BCVA in the OS improved to 0.2. Fundus examination showed a vasoproliferative lesion in the inferotemporal area, with accompanying nasal fibrosis extending from the optic disc and small hemorrhages on the surface of the lesion. A decision was made to include cryotherapy. During subsequent follow-up visits, progression of exudative and hemorrhagic changes was observed, including the presence of hard exudates and vitreous hemorrhage. Despite periodic stabilization of the clinical picture, new intraretinal fluid spaces appeared.

**Figure 3:**
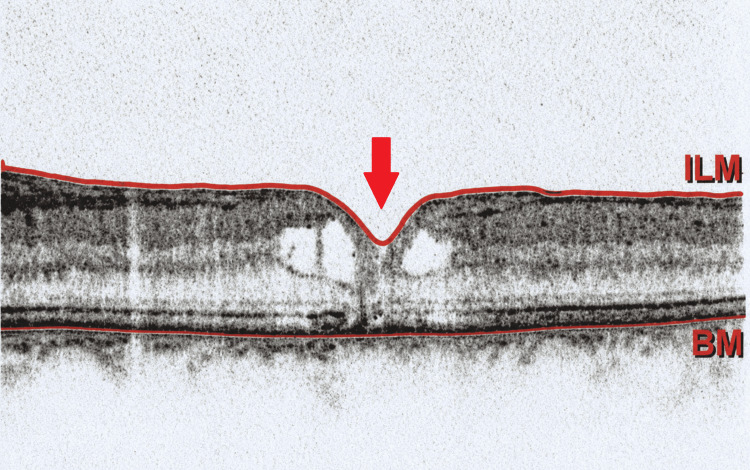
Optical coherence tomography of the left eye showing increased retinal thickening with a reduced amount of intraretinal and subretinal fluid (arrow).

In the further course of the disease, ophthalmoscopy showed dilation of the vessels in the inferotemporal quadrant. Although OCT showed a temporary reduction in the amount of intraretinal fluid, the course of the disease remained unstable (Figure 3).

**Figure 4:**
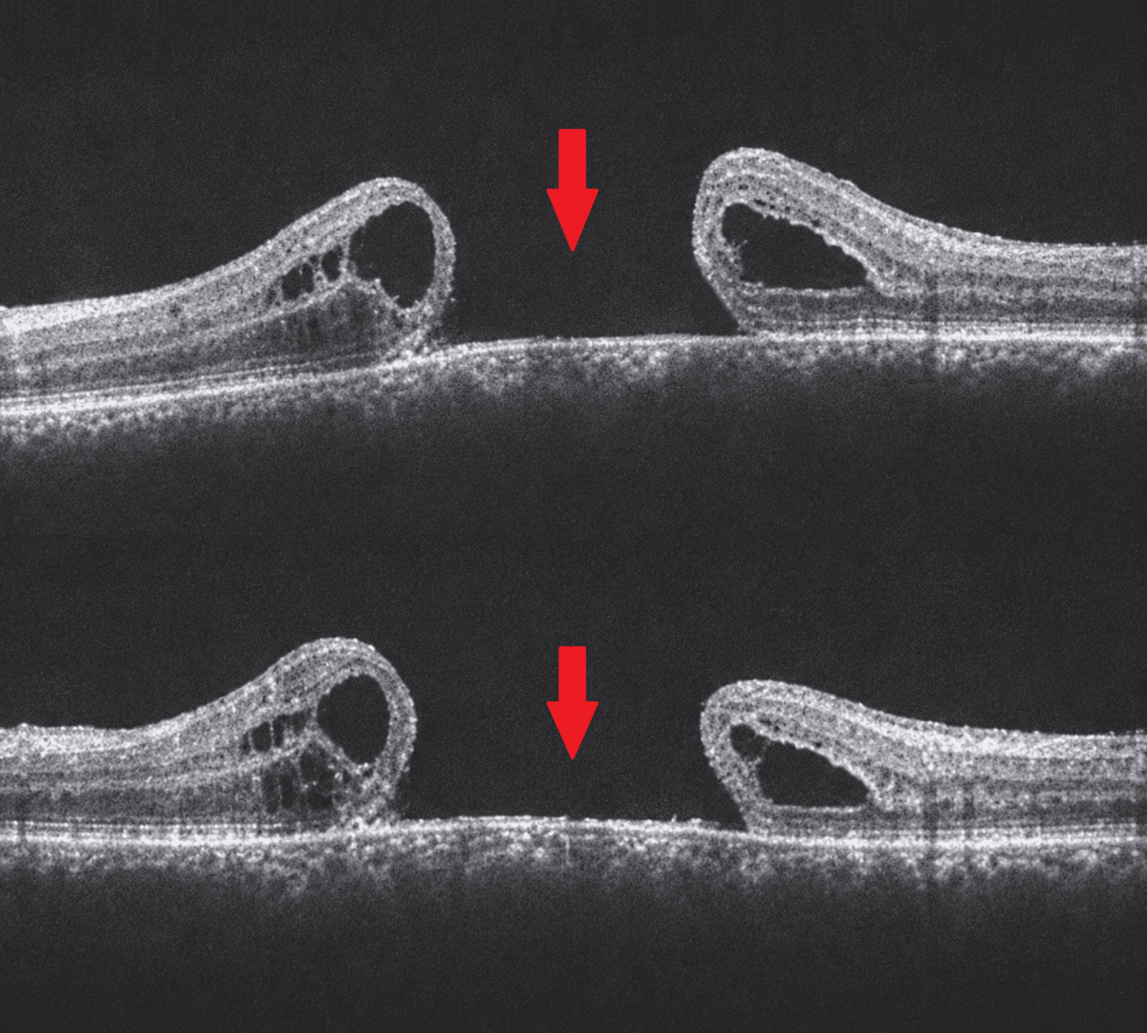
Optical coherence tomography (OCT) of the left eye (OS) 11 months after treatment showing the formation of a full-thickness macular hole (FTMH) (arrows).

After several months, anterior segment examination of the OS revealed early signs of band keratopathy in the inferior part of the cornea, suggesting a chronic course of the disease. A subsequent OCT examination showed significant deterioration, with retinal edema and the formation of a full-thickness macular hole (FTMH), while the retina remained fully attached (Figure 4).

**Figure 5:**
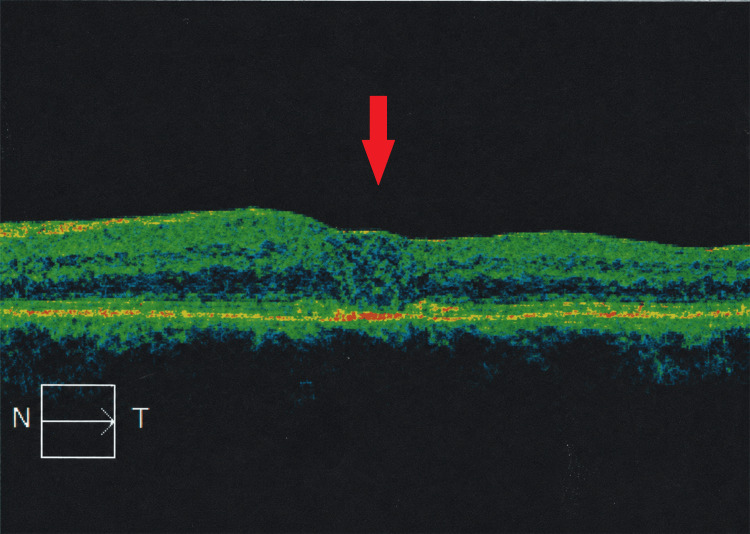
Optical coherence tomography (OCT) of the left eye (OS) one month after pars plana vitrectomy (PPV). OCT of the macula demonstrating closure of the full-thickness macular hole after PPV. Residual irregularity of the foveal contour is visible (arrow).

Due to the progression of retinal changes despite conservative treatment, the patient was qualified for surgical management. Pars plana vitrectomy (PPV) of the OS was performed, together with laser treatment of pathological vessels in the peripheral retina. One month after surgery, postoperative imaging showed closure of the FTMH, which was visible on the OCT (Figure 5). Considering the clinical course, Coats disease was diagnosed.

## Discussion

As mentioned in the Introduction, Coats disease is a non-hereditary vascular disorder of the retina. It is characterized by intraretinal or subretinal exudation and retinal telangiectasia resulting from idiopathic endothelial dysfunction. The clinical course includes a wide spectrum of findings, ranging from abnormalities of single vessels without exudation to massive lipid-rich exudates. The disease is rare, most commonly affects young males, and usually involves only one eye [1,2,8].

Coats disease differs phenotypically depending on the age at onset. In the pediatric population, the disease usually appears between six and eight years of age and has a progressive course. Vascular abnormalities are mainly located in the retinal capillaries of the temporal quadrants, between the equator and the ora serrata [8]. In adults, Coats disease most often begins after the age of 35. These patients are commonly diagnosed with diabetes mellitus, arterial hypertension, or hypercholesterolemia. The rate of progression in this population is slower; the lesions are located in the perimacular and peripheral retina, and the risk of retinal detachment is lower than in children [2].

Coats disease shows clear clinical differences between the pediatric and adult forms. In children, the disease has a more aggressive course. A typical symptom is leukocoria, which is often the first sign of the disease and results from extensive retinal exudation. The lesions involve both the macular and peripheral retina, and the exudates are diffuse. Retinal telangiectasia is common and is accompanied by marked disruption of the vascular barrier. In this group of patients, there is a high risk of exudative retinal detachment and a relatively high probability of neovascular glaucoma, reflecting a more severe and advanced course of the disease [8,9].

In adults, the disease is much milder and progresses more slowly. Leukocoria is practically absent, and the symptoms are less striking and more clinically subtle. The lesions are mainly located in the perimacular and peripheral retina, and the exudates are more often focal than diffuse. Telangiectasia is less common, while intraretinal and preretinal hemorrhages are observed more frequently. The risk of retinal detachment and neovascular glaucoma is much lower than in children. In addition, systemic diseases such as diabetes mellitus, hypertension, and hypercholesterolemia are more common in adults [2,8,9].

In the 39-year-old patient described in this report, the dominant symptom was sudden unilateral deterioration of visual acuity. Fundus examination showed focal exudation in the inferotemporal quadrant, intraretinal and preretinal hemorrhages, and extravasation into the vitreous body. The clinical picture of this patient was more similar to the adult form of Coats disease because of the age at onset, absence of leukocoria, focal nature of the exudates, and presence of a hemorrhagic component. However, the rapid progression of the disease, the presence of telangiectasia, and the absence of predisposing systemic diseases were not typical for this age group.

The diagnosis of Coats disease is based on a detailed ophthalmological examination and the use of multimodal imaging techniques, which allow both confirmation of the diagnosis and assessment of disease severity [7]. Fluorescein angiography plays a key role in imaging diagnosis, as it enables the assessment of leakage from abnormal vessels and the identification of areas of retinal ischemia [10]. OCT and OCT angiography (OCTA) are also used, as they allow detailed structural assessment of the retina and monitoring of disease activity. Ocular ultrasonography and wide-field fundus imaging are also useful in the diagnostic process, especially in advanced cases or when visualization of retinal structures is limited [7].

In adults, the diagnosis of Coats disease is a diagnosis of exclusion. Therefore, other causes of telangiectasia and exudative retinal detachment must be ruled out. In particular, secondary “Coats-like” changes associated with retinal vascular diseases, such as diabetic retinopathy or central retinal vein occlusion, should be excluded. It is also important to differentiate the disease from inflammatory and infectious retinal disorders and from post-traumatic complications, which may present with a similar clinical picture of exudation and retinal detachment. In contrast to the pediatric population, differentiation from retinoblastoma is less often required in adults; however, in atypical cases, neoplastic processes should still be included in the differential diagnosis [11]. In addition, systemic diseases involving the retinal vessels should be excluded [12]. In our patient, the above possible differential diagnoses were ruled out.

Shields proposed a five-stage classification system intended to help select the most appropriate treatment method. This system is based on treatment outcomes observed after the introduction of a given therapeutic approach [13]. Stage 1 is characterized by telangiectasia without exudation. In stage 2, telangiectasia is accompanied by exudation, which may be located outside the fovea (2A) or involve the fovea (2B). Stage 3 includes exudative retinal detachment, which may be subtotal and extrafoveal (3A1), subtotal with foveal involvement (3A2), or total (3B). Stage 4 is characterized by total retinal detachment with secondary glaucoma. Stage 5 represents advanced end-stage disease with total retinal detachment, often accompanied by cataract and progression to phthisis bulbi [14]. Over time, this classification was modified. Daruich et al. proposed a classification system introducing two additional subcategories within stage 2B: stage 2B1, without subfoveal nodules, and stage 2B2, with subfoveal nodules [15].

In our patient, the initial Shields classification changed over the course of the disease. The patient was initially classified as stage 2B1 because of telangiectasia in the inferotemporal quadrant and intraretinal exudation. After laser therapy, follow-up examinations revealed a vasoproliferative tumor, changing the stage to 2B2. One year after the initial diagnosis, the patient developed an FTMH. However, the retina remained fully attached, and therefore the preoperative classification remained unchanged.

The choice of treatment depends on the disease stage according to the classification developed by Shields et al. Patients without exudation or with minimal exudation may be observed. Laser photocoagulation is used in stages 1-3A when exudation is present. Its aim is to ablate the existing telangiectatic vessels. Cryotherapy may also be used as an alternative. In advanced retinal detachments in stages 3 and 4, vitreoretinal surgery may be used when retinal reattachment is possible. When the eye is no longer functional, blind, and painful, enucleation is performed [1].

Intravitreal anti-VEGF therapy may also be used during treatment because of high intraocular expression of VEGF. It has been confirmed that combined treatment, including anti-VEGF therapy, laser coagulation, and cryotherapy, may improve the prognosis in patients with reduced visual acuity [6]. In our patient, laser therapy and cryotherapy were performed, and the treatment was supplemented with intravitreal bevacizumab injections. Despite the escalation of treatment, follow-up showed the development of an FTMH. Therefore, PPV was performed. The internal limiting membrane (ILM) was peeled, leaving a central remnant. At the end of the procedure, sulfur hexafluoride (SF6) gas was injected. This treatment resulted in closure of the FTMH, which was shown on OCT during postoperative follow-up.

## Conclusions

Coats disease is a non-hereditary disorder of idiopathic etiology. It typically occurs in male children and is characterized by a rapid clinical course. In adults, it is diagnosed less frequently and usually progresses more slowly. The case of our patient differs from the typical presentation of Coats disease in adults. Despite the use of non-surgical treatment methods, disease progression occurred. This case shows that conservative treatment may be insufficient in the management of Coats disease in adult patients.
